# Preservation of renal endothelial integrity and reduction of renal edema by aprotinin does not preserve renal perfusion and function following experimental cardiopulmonary bypass

**DOI:** 10.1186/s40635-021-00393-9

**Published:** 2021-06-25

**Authors:** Nicole A. M. Dekker, Anoek L. I. van Leeuwen, Matijs van Meurs, Jill Moser, Jeannette E. Pankras, Nicole N. van der Wel, Hans W. Niessen, Marc G. Vervloet, Alexander B. A. Vonk, Peter L. Hordijk, Christa Boer, Charissa E. van den Brom

**Affiliations:** 1 Department of Anesthesiology, Experimental Laboratory for Vital Signs, Amsterdam Cardiovascular Sciences, Amsterdam UMC, Vrije Universiteit, Amsterdam, The Netherlands; 2 Department of Cardiothoracic Surgery, Amsterdam Cardiovascular Sciences, Amsterdam UMC, Vrije Universiteit, Amsterdam, The Netherlands; 3 Department of Physiology, Amsterdam Cardiovascular Sciences, Amsterdam UMC, Vrije Universiteit, Amsterdam, The Netherlands; 4 Department of Pathology and Medical Biology, University Medical Center Groningen, Groningen, The Netherlands; 5 Department of Critical Care Medicine, University Medical Center Groningen, Groningen, The Netherlands; 6 Department of Medical Biology, Electron Microscopy Centre Amsterdam, Amsterdam UMC, University of Amsterdam, Amsterdam, The Netherlands; 7 Department of Pathology, Amsterdam Cardiovascular Sciences, Amsterdam UMC, Vrije Universiteit, Amsterdam, The Netherlands; 8 Department of Nephrology, Amsterdam Cardiovascular Sciences, Amsterdam UMC, Vrije Universiteit, Amsterdam, The Netherlands; 9 Department of Intensive Care Medicine, Laboratory for Experimental Intensive Care and Anesthesiology (LEICA), Amsterdam UMC, University of Amsterdam, Amsterdam, The Netherlands

**Keywords:** Cardiopulmonary bypass, Microcirculation, Capillary permeability, Acute kidney injury, Renal perfusion, Edema

## Abstract

**Background:**

Acute kidney injury is a severe complication following cardiopulmonary bypass (CPB) and is associated with capillary leakage and microcirculatory perfusion disturbances. CPB-induced thrombin release results in capillary hyperpermeability via activation of protease-activated receptor 1 (PAR1). We investigated whether aprotinin, which is thought to prevent thrombin from activating PAR1, preserves renal endothelial structure, reduces renal edema and preserves renal perfusion and reduces renal injury following CPB.

**Methods:**

Rats were subjected to CPB after treatment with 33.000 KIU/kg aprotinin (*n* = 15) or PBS (*n* = 15) as control. A secondary dose of 33.000 KIU/kg aprotinin was given 60 min after initiation of CPB. Cremaster and renal microcirculatory perfusion were assessed using intravital microscopy and contrast echography before CPB and 10 and 60 min after weaning from CPB. Renal edema was determined by wet/dry weight ratio and renal endothelial structure by electron microscopy. Renal PAR1 gene and protein expression and markers of renal injury were determined.

**Results:**

CPB reduced cremaster microcirculatory perfusion by 2.5-fold (15 (10–16) to 6 (2–10) perfused microvessels, *p* < 0.0001) and renal perfusion by 1.6-fold (202 (67–599) to 129 (31–292) au/sec, *p* = 0.03) in control animals. Both did not restore 60 min post-CPB. This was paralleled by increased plasma creatinine (*p* < 0.01), neutrophil gelatinase-associated lipocalin (NGAL; *p* = 0.003) and kidney injury molecule-1 (KIM-1; *p* < 0.01). Aprotinin treatment preserved cremaster microcirculatory perfusion following CPB (12 (7–15) vs. 6 (2–10) perfused microvessels, *p* = 0.002), but not renal perfusion (96 (35–313) vs. 129 (31–292) au/s, *p* > 0.9) compared to untreated rats. Aprotinin treatment reduced endothelial gap formation (0.5 ± 0.5 vs. 3.1 ± 1.4 gaps, *p* < 0.0001), kidney wet/dry weight ratio (4.6 ± 0.2 vs. 4.4 ± 0.2, *p* = 0.046), and fluid requirements (3.9 ± 3.3 vs. 7.5 ± 3.0 ml, *p* = 0.006) compared to untreated rats. In addition, aprotinin treatment reduced tubulointerstitial neutrophil influx by 1.7-fold compared to untreated rats (30.7 ± 22.1 vs. 53.2 ± 17.2 neutrophil influx/section, *p* = 0.009). No differences were observed in renal PAR1 expression and plasma creatinine, NGAL or KIM-1 between groups.

**Conclusions:**

Aprotinin did not improve renal perfusion nor reduce renal injury during the first hour following experimental CPB despite preservation of renal endothelial integrity and reduction of renal edema.

**Supplementary Information:**

The online version contains supplementary material available at 10.1186/s40635-021-00393-9.

## Background

Acute kidney injury (AKI) is one of the most serious complications following cardiac surgery with cardiopulmonary bypass (CPB). AKI occurs in 2 to 40% of the patients depending on studied population and classification used [1] and is associated with prolonged hospitalization and postoperative morbidity [2]. AKI is also considered as strong risk factor for mortality after cardiac surgery with mortality rates up to 70% in patients with AKI requiring dialysis [3]. Despite its well-known impact on postoperative outcome and health care costs [4], therapeutic strategies to effectively prevent or treat AKI remain to be identified.

To date, AKI following cardiac surgery with CPB is mainly ascribed to renal hypoperfusion and renal hypoxia [5, 6]. Previously, we showed that CPB induces microcirculatory perfusion disturbances, [7] which remain disturbed for at least three days postoperatively [8]. The etiology of these microcirculatory perfusion disturbances and AKI are multifactorial [9]. For example, CPB-associated hemodilution and changes in vascular resistance reduce renal oxygen delivery [10, 11]. In addition, initiation of CPB induces a systemic inflammatory response [12], resulting in renal neutrophil influxes and renal edema following capillary leakage [13]. In particular, renal capillary leakage and edema formation seem to play a central role in the development of microcirculatory perfusion disturbances and AKI. Previous results from our group revealed that therapeutic inhibition of capillary leakage improved cremaster microcirculatory perfusion and renal function following experimental CPB [14, 15].

Besides inducing a systemic inflammatory response, CPB is associated with the generation of thrombin, which is an independent risk factor for the development of AKI [16]. Thrombin can activate a wide range of receptors, but has extremely high affinity for protease-activated receptor 1 (PAR1) which is present on both platelets and endothelial cells [17, 18]. As a result, thrombin generation following CPB is associated with activation of coagulation and increased vascular permeability through endothelial activation. These results suggest that thrombin release could contribute to CPB-associated capillary leakage and AKI and therefore prevention of thrombin-induced activation of PAR1 could be of interest to protect renal function during cardiac surgery with CPB [19].

Aprotinin is a serine protease inhibitor associated with anti-inflammatory and anti-fibrinolytic properties during cardiac surgery [20, 21]. Despite the controversy regarding its effect during cardiac surgery on postoperative morbidity, aprotinin exerts a variety of interesting mechanistic actions, which have scarcely been investigated and may be of additional interest for its use during cardiac surgical procedures. Although aprotinin was initially introduced for clinical use in cardiac surgery patients to prevent blood loss and preserve platelet function, it has also been shown to prevent thrombin from cleaving and activating PAR1 on both platelets [21, 22] and endothelial cells [23, 24]. Therapeutically targeting the thrombin/PAR1 pathway may provide a unique treatment strategy to reduce both the pro-thrombotic and pro-inflammatory state to preserve organ function following CPB [19]. Therefore, we investigated whether aprotinin reduces renal edema and thereby protects renal perfusion and renal function following experimental CPB.

## Materials and methods

### Animals

All procedures were approved by the Institutional Animal Care and Use Committee of the VU University, the Netherlands (Animal welfare number: AVD1140020172144; local protocol number: 2144-ANES 17-01), and conducted following the EU Directive (2010/63EU) on the protection of vertebrate animals used for experimental and other scientific purposes and are reported in accordance with the ARRIVE guidelines on animal research. [25].

**Fig. 1:**
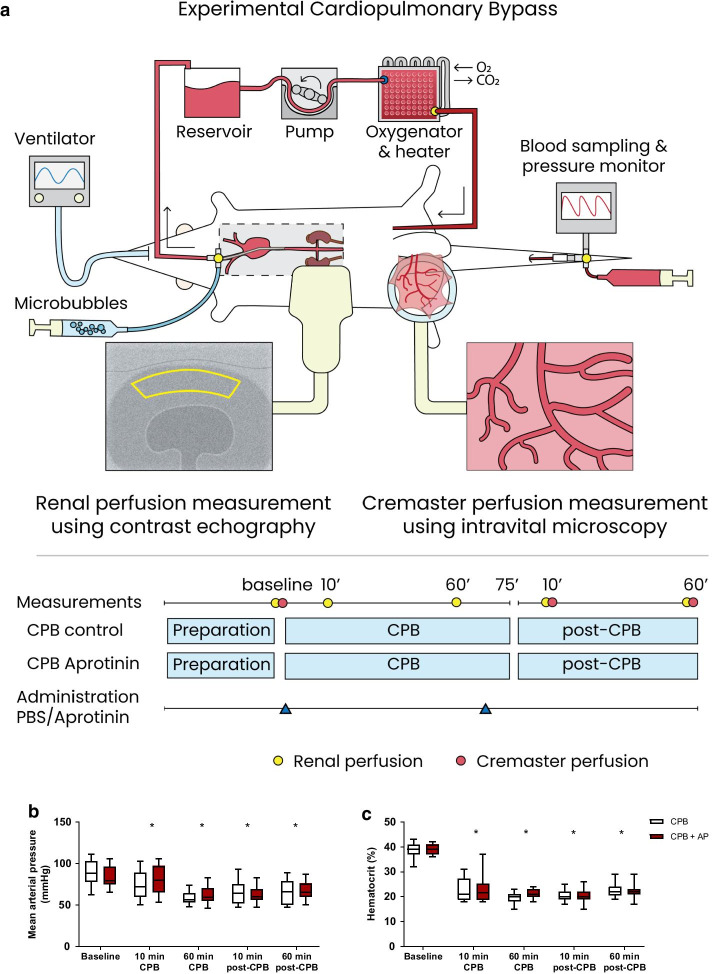
Schematic overview and perioperative hemodynamics. Rats were randomized to undergo cardiopulmonary bypass with PBS (CPB control, *n* = 15) or cardiopulmonary bypass with aprotinin treatment (CPB + AP, *n* = 15). AP or PBS were administered after baseline measurements were performed and a secondary dose was given 60 min after onset of CPB. Microcirculatory perfusion measurements in the cremaster muscle (yellow circles) were performed directly after the surgical preparation before onset of CPB (baseline), 10 min after initiation of CPB (10 min CPB), 60 min after initiation of CPB (60 min CPB), 10 min after weaning from CPB (10 min post-CPB) and 60 min after weaning from CPB (60 min post-CPB). Renal perfusion measurements (red circles) were performed before onset of CPB (baseline), 10 min after weaning from CPB (10 min post-CPB) and 60 min after weaning from CPB (60 min post-CPB). Plasma was collected at baseline, 60 after initiation of CPB (60 min CPB) and 60 min after weaning from CPB (60 min post-CPB). Rats were culled 60 min after weaning from CPB and urine samples and kidneys were stored for further analyses (**a**). Mean arterial pressure (**b**) and hematocrit levels (**c**) in rats during and following cardiopulmonary bypass (CPB; white boxes; *n* = 15) or CPB with aprotinin treatment (CPB + AP; red boxes; *n* = 15). Boxes and whiskers represent median, interquartile and full range, *p < 0.05 CPB vs. CPB baseline, #*p* < 0.05 CPB vs. CPB + AP

Male Wistar rats of 375–425 g (Charles River Laboratories, Brussels, Belgium) were randomly assigned to undergo cardiopulmonary bypass (CPB) with aprotinin treatment (CPB + AP, *n* = 15) or phosphate buffered solution (PBS) as control (CPB, *n* = 15) for measurement of cremaster and renal perfusion (Fig. 1a). Rats were culled by blood withdrawal under 5.0% isoflurane inhalation 60 min after weaning from CPB. Blood was protracted from the femoral arterial line in approximately 1 min using a 10 mL syringe. Kidneys were isolated and blood and urine samples were collected and stored at − 80 °C for additional molecular analyses.

An additional group of sham rats (Sham, *n* = 9) were included to investigate the effect of CPB on renal outcome parameters. The sham procedure was identical to the CPB groups except for initiation and weaning of CPB, but including heparin, rocuronium bromide and protamine administration.

### Aprotinin treatment

All tubes containing either aprotinin or PBS were prepared at the start of the study and randomly numbered. Aprotinin (Trasylol®, Nordic Pharma, Baarn, The Netherlands) was dissolved in PBS and 33.000 KIU/kg was administered via the jugular vein 20 min prior to CPB and a secondary dose of 33.000 KIU/kg was given 60 min after initiation of CPB. Equal volumes of PBS were administered in control rats. Investigators performing the experiments were blinded for group allocation. Group allocation was revealed when data acquisition and analyses were completed.

### Anesthesia and surgical preparation

All animals were anesthetized as previously reported [14, 15] with 4.0% isoflurane (Ivax Farma, Haarlem, The Netherlands) in oxygen. Following endotracheal intubation with a 16G catheter (Venflon Pro, Becton Dickinson, Helsingborg, Sweden), lungs were mechanically ventilated (UMV-03, UNO Roestvaststaal BV, Zevenaar, The Netherlands; PEEP 2–4 cm H_2_O, respiratory rate of 60–65 breaths/min, tidal volume ~ 10 ml/kg) and anesthesia was maintained with 1.5–2.0% isoflurane in oxygen-enriched air (40% O_2_/ 60% N_2_). Additionally, fentanyl boluses (12 µg/kg) were administered every 30–40 min. Respiratory rate was adjusted based on blood gas values to maintain pH and partial pressure of carbon dioxide within physiological limits. Depth of anesthesia was continuously monitored and adjusted if necessary based on heart rate and mean arterial pressure. A 22G catheter (Venflon Pro, Becton Dickinson, Helsingborg, Sweden) was placed in the caudal (tail) artery for continuous measurements of arterial blood pressure and blood withdrawal for blood gas analysis and hematocrit measurements (ABL80, radiometer, Copenhagen, Denmark). Arterial blood pressure, ECG and heart rate were continuously recorded using PowerLab software (PowerLab 8/35, Chart 8.0; AD Instruments Pty, Ltd., Castle Hill, Australia).

The left cremaster muscle was isolated and prepared for cremaster perfusion measurements as previously described [14, 15]. Heparin (500 IU/kg, LEOPharma, Amsterdam, The Netherlands) was administered followed by cannulation of the right femoral artery with a 20G catheter (Arterial Cannula, Becton Dickinson, Helsingborg, Sweden) for arterial inflow of the CPB circuit. The right jugular vein was catheterized with a 20G catheter (Venflon Pro, Becton Dickinson, Helsingborg, Sweden) for administration of aprotinin or PBS, and the administration of microbubbles for contrast enhanced echography (CEUS). Further information can be found in the Additional file 1.

### Cardiopulmonary bypass

The protocol for CPB was performed as previously described [14, 15]. In summary, the CPB circuit consisted of an open venous reservoir, a roller pump (Pericor SF70, Verder, Haan, Germany), and an oxygenator-heat exchanger with a three-layer hollow fiber membrane for gas exchange (Ing. M. Humbs, Valley, Germany) (Fig. 1). A 1.0-mm-diameter arterial line (LectroCath, Vygon, Ecouen, France) was connected to the femoral inflow catheter. The circuit was primed with 10 ml of 6% hydroxyethyl starch (HES; Voluven, Fresenius Krabi, Halden, Norway). During CPB, CO_2_ and O_2_ pressures of the oxygenator membrane of the CPB circuit were adjusted based on blood gas values to maintain pH and partial pressure of carbon dioxide within physiological limits. Additional doses of 6% hydroxyethyl starch (1 mL bolus; 6%HES; Voluven, Fresenius Krabi, Halden, Norway) were administrated when necessary to maintain target CPB flow rates > 150 ml/kg/min. Hematocrit levels were checked to avoid excessive fluid resuscitation. Moreover, boluses of phenylephrine (0.1 mg) were administered if necessary to maintain mean arterial pressure above 50 mmHg to sustain organ perfusion pressure. Detailed information can be found in the Additional file 1.

### Cremaster microcirculatory perfusion

Microcirculatory perfusion measurements were performed using a 10 × objective on an intravital microscope (AxiotechVario 100HD, Zeiss, Oberkochen, Germany) connected to a digital camera (scA640, Basler, Ahrensburg, Germany) with a final magnification of 640x, as described previously [14, 15]. Briefly, three regions of the microvasculature (vessels up to 25 µm diameter) in the cremaster muscle with adequate perfusion quality were selected during baseline. These exact predefined regions were followed throughout the experiment: after surgical preparation of the cremaster muscle before onset of CPB (baseline), 10 min after initiation of CPB (10 min CPB), 60 min after initiation of CPB (60 min CPB), 10 min after weaning from CPB (10 min post-CPB) and 60 min after weaning from CPB (60 min post-CPB) (Fig. 1a).

Microcirculatory perfusion analyses were performed offline by an investigator who was blinded for treatment allocation. Analyses were performed as previously described by categorizing capillaries based on flow patterns as follows: perfused (continuous blood flow), intermittently perfused (blood flow was reversed or arrested for at least 50% of the time) and non-perfused capillaries (no flow). Pearson coefficient of the intra- and inter-observer correlation of the number of perfused vessels were 0.982 (*p* < 0.0001) and 0.959 (*p* < 0.0001), respectively.

### Renal perfusion

Contrast enhanced echography was performed as previous described [26] with a Vevo 2100 Imaging System and MS 250 Nonlinear Contrast Imaging transducer (VisualSonics Inc, Toronto, Canada). Microbubbles were continuously infused via the jugular vein with a rate of 150 µl/min and the right kidney was visualized in the longitudinal plane before onset of CPB (baseline), 10 min after weaning from CPB (10 min post-CPB) and 60 min after weaning from CPB (60 min post-CPB) (Fig. 1).

For each measurement, regions of interest were drawn in the renal cortex by an investigator who was blinded for treatment allocation. Renal signal intensities were fitted (*Y* = *Y*0 + (*A* − *Y*0) ⋅ (1 − exp^(−*β*⋅*x*)^)) for calculation of renal vascular blood volume (*A*) and renal vascular filling velocity (*β*), which corresponds to renal blood exchange rate. The estimate of renal perfusion was calculated as the product of *A* and *β*. Detailed information can be found in the Additional file 1.

### Renal edema

Renal tissue was harvested at the end of the experiment under terminal anesthesia. Wet tissue was weighed and dried at 70 °C. After 24 h, dry tissue was weighed and renal wet-to-dry weight (W/D) ratio was calculated as estimate for tissue water content.

### Electron microscopy

Renal tissue (*n* = 3–4 per group) was used for analysis of renal cortical capillary endothelial ultrastructure [14]. Renal tissue was harvested at the end of the experiment and immediately fixed in Karnovsky fixative. Ten random capillaries in the renal cortex were captured using electron microscope imaging (Tecnai 12G^2^, Fei company, with a VALETA side entry camera) by an investigator blinded to group allocation. Subsequently, images were analyzed for signs of endothelial injury and activation, including luminal membrane blebs, vacuoles, endothelial gaps and endothelial detachment. Detailed information can be found in the Additional file 1.

### Plasma and urinary analyses

Arterial blood was collected in EDTA tubes at baseline, 60 after initiation of CPB (60 min CPB), and 60 min after weaning from CPB (60 min post-CPB) and urine samples were obtained one hour after weaning of CPB (60 min post-CPB). Urinary levels of neutrophil gelatinase-associated lipocalin (NGAL) and kidney injury molecule-1 (KIM-1) and plasma levels of NGAL, KIM-1, creatinine, thrombin–antithrombin complex (TAT), soluble thrombomodulin (sTM), von Willebrand Factor (vWF) and interleukin-6 (IL-6) were measured with ELISA (Cloud-Clone Corporation, Wuhan, Hubei, China) in accordance to the manufacturer. Detailed information can be found in the Additional file 1.

### Immunohistochemical analyses

Renal tissue was fixed in 4% formalin and embedded in paraffin. Immunohistochemical staining was performed for assessment of glomerular and tubulointerstitial neutrophil infiltration by anti-myeloperoxidase (MPO; 1:50, Abcam, Cambridge, UK) [14, 27] and for assessment of fibrin-stained thrombi deposition by Martius, Scarlet and Blue (MSB) staining. The investigator performing the analysis was blinded to group allocation. Detailed information can be found in the Additional file 1.

### Gene expression

Total RNA was extracted from 10–30 mg frozen kidney tissue and isolated using the RNeasy mini kit (Qiagen, Venlo, The Netherlands), as previously described [15]. The following genes were used for quantitative PCR: *KIM-1, NGAL, ICAM-1, VCAM-1, E-selectin, P-selectin* and *PAR1* (Applied Biosystems, Foster City, CA). ΔcT was calculated and mRNA expression levels were normalized to the housekeeping gene Tfrc.

### Protein expression

Frozen kidney tissue was homogenized to obtain cellular protein fractions for western blot analysis as described previously [15, 26]. Protein expression of uncleaved PAR1 was analyzed using anti-PAR1/Thrombin Receptor (25 µg protein, 1:200, ab32611, Abcam, USA). Immunoblots were quantified by densitometric analysis of films (ImageQuant TL, v8.1, GE Healthcare, USA). Signal was normalized to glyceraldehyde 3-phosphate dehydrogenase protein expression (GAPDH; 1:5000, No. 2118, Cell Signaling Technology, USA).

### Statistical analysis

All data are expressed as median (full range) or mean ± standard deviation (SD) and analyzed using GraphPad Prism 8.0 (GraphPad Software, La Jolla, CA, USA). Sample size was calculated based on previous rat experiments in which cardiopulmonary bypass induced a decrease in cremaster perfused capillaries from 9.2 to 5.5 ± 1.5 perfused vessels per recording [14, 15]. Sample size for renal perfusion measurements were based on pilot experiments in which cardiopulmonary bypass induced a decrease in renal perfusion from of 95 to 48 ± 12. Using an alpha of 0.05 and a power of 0.90, a sample size of 8 per group was required for cremaster perfusion measurements and a sample size of 15 was required for renal measurements. Normality of distribution was tested with the Shapiro–Wilk test. Time-dependent differences were analyzed with two-way ANOVA with repeated measures and Bonferroni post hoc analysis. Two-sided Mann–Whitney *U*- and Wilcoxon tests were used to evaluate differences in-between groups and within groups, respectively. *p* values < 0.05 were considered as statistically significant.

## Results

### Hemodynamics and blood gas analysis

All animals completed the experimental protocol. Rats weighed 400 ± 8 and 401 ± 10 g for control and aprotinin-treated groups, respectively (*p* = 0.71). Cardiopulmonary bypass was associated with reduced mean arterial pressure (Fig. 1b, median (full range), 88 (62–111) to 72 (50–103) mmHg, *p* = 0.02) and hematocrit levels (Fig. 1c, 39 (36–42) to 22 (18–37) %, *p* < 0.0001), which remained altered in the first hour after weaning from CPB (*p* = 0.0001 and *p* < 0.0001, respectively). Moreover, CPB resulted in a reduction in heart rate (375 (343–408) to 320 (257–371) bpm, *p* = 0.0001) and temperature (36.5 (35.3–37.7) to 35.0 (33.4–35.7) °C, *p* < 0.0001). Also, onset of CPB reduced bicarbonate levels (24 (19–27) to 18 (14–21) *p* < 0.0001) and a reduction in pH was observed immediately after weaning from CPB (7.4 (7.3–7.5) to (7.2 (7.1–7.4), *p* < 0.0001), whereas oxygen saturation and partial pressures of carbon dioxide and oxygen remained unaltered.

Aprotinin did not affect mean arterial pressure or hematocrit levels throughout the experiment (Fig. 1b, c). Moreover, pH (*p* = 0.6), base excess (*p* > 0.9), partial pressures of carbon dioxide (*p* > 0.9) and oxygen (*p* > 0.9) were comparable between CPB controls and aprotinin-treated animals. There were no differences in the use of isoflurane anesthesia (*p* = 0.71), phenylephrine (*p* = 0.63), CPB flow rates (*p* > 0.9) or body temperature (*p* = 0.64) between groups (data not shown).

### Cremaster perfusion

**Fig. 2:**
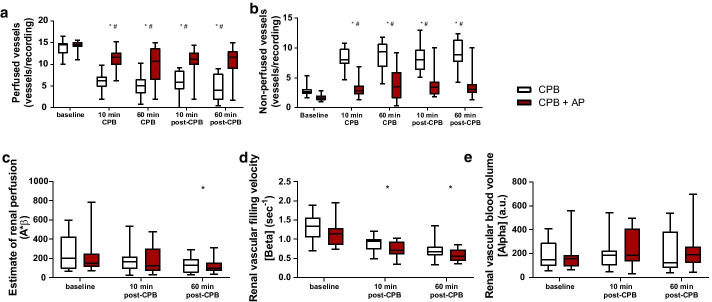
Microcirculatory perfusion following CPB. Continuously perfused vessels (**a**) and non-perfused vessels (**b**) measured in rat cremaster muscle using intravital microscopy. Renal blood volume (**c**), renal vascular filling velocity (**d**), and estimate of renal perfusion (**e**) measured in the right renal cortex using contrast enhanced ultrasound in rats undergoing cardiopulmonary bypass (CPB; white boxes; *n* = 15) or rats undergoing CPB with aprotinin treatment (CPB + AP; red boxes; *n* = 15). Boxes and whiskers represent median, interquartile and full range, *p < 0.05 CPB vs. CPB baseline, #*p* < 0.05 CPB vs. CPB + AP

Onset of CPB was associated with a 2.5-fold reduction in the number of perfused vessels (15 (10–16) to 6 (2–10) perfused vessels per recording, *p* < 0.0001; Fig. 2a), and a 2.6-fold increase in the number of non-perfused vessels (3 (2–5) to 8 (5–11) non-perfused vessels per recording, *p* < 0.0001; Fig. 2b) compared with baseline measurements in CPB control rats. Microcirculatory perfusion remained impaired in the first hour after weaning from CPB. The number of intermittently perfused vessels remained stable throughout the experiment (0 (0–0) to 0 (0–0) intermittently perfused vessels per recording, baseline to 60 min post-CPB, *p* > 0.9).

In aprotinin-treated animals, onset of CPB was associated with a 1.5-fold reduction in the number of perfused vessels (15 (11–16) to 12 (7–15) perfused vessels per recording, *p* = 0.0002; Fig. 2a) and a 1.5-fold increase in number of non-perfused vessels (2 (1–3) to 3 (1–7) non-perfused vessels per recording, *p* = 0.0002; Fig. 2b). This reduction was less profound compared to untreated CPB animals (15 (11–16) to 12 (7–15) vs. 15 (10–16) to 6 (2–10) perfused vessels per recording, CPB + AP vs. CPB, *p* < 0.0001; 2 (1–3) to 3 (1–7) vs. 3 (2–5) to 8 (5–11) non-perfused vessels per recording, CPB + AP vs. CPB, *p* < 0.0001). No differences in baseline microcirculatory perfusion nor intermittently perfused vessels were observed between groups.

### Renal perfusion

CPB did not affect renal blood volume (*A*) in control animals throughout the study period (Fig. 2c, 149 (56–410) vs. 123 (38–540) a.u., *p* > 0.9, 60 min post-CPB vs. baseline). However, a reduction in renal vascular filling velocity (*β*) was observed immediately after weaning from CPB compared with baseline measurements (1.3 (0.7–1.9) to 0.9 (0.5–1.2) per sec, *p* < 0.0001), and remained decreased one hour after weaning from CPB (Fig. 2d, 0.7 (0.4–1.4), *p* < 0.0001, 60 min post-CPB). Overall, CPB reduced renal perfusion one hour after weaning from CPB compared with baseline measurements in untreated CPB animals (Fig. 2e, 202 (67–599) to 129 (31–292) a.u./sec, *p* = 0.03, baseline to 60 min post-CPB).

Aprotinin treatment did not affect renal blood volume (*p* > 0.9), filling velocity (*p* = 0.52) nor renal perfusion (*p* > 0.9) throughout the course of the experiment compared with untreated CPB animals (Fig. 2). Estimate of renal perfusion was significantly reduced in both CPB groups compared to sham animals 60 min post-CPB (237 (197–645) vs. 129 (31–292) a.u./s, sham vs CPB control, *p* = 0.044; (237 (197–645) vs. 96 (35–520) a.u./ sec, sham vs CPB + AP, *p* = 0.03, RM ANOVA (Additional file 2: Figure S2)).

### Renal edema and fluid requirements

**Fig. 3:**
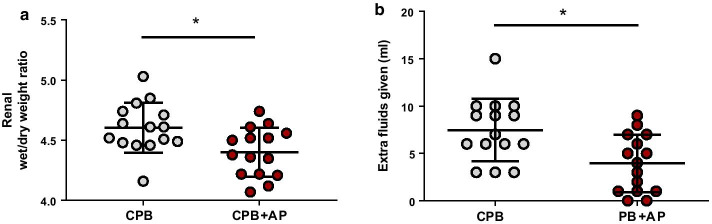
Renal edema and fluid requirements. Renal wet/dry weight ratio (**a**) and fluid requirements (**b**) in rats undergoing cardiopulmonary bypass (CPB; grey circles; *n* = 15) or CPB with aprotinin treatment (CPB + AP; red circles; *n* = 15). Data are presented as mean ± standard deviation, *p < 0.05 CPB vs. CPB + AP

Renal wet/dry weight ratio was reduced in rats treated with aprotinin compared to untreated CPB rats one hour after weaning from CPB (Fig. 3b, 4.4 ± 0.2 vs. 4.6 ± 0.2, CPB + AP vs. CPB, *p* = 0.01), indicative of reduced edema formation. Aprotinin-treated rats required 50% less fluids during and after CPB compared with untreated CPB animals (Fig. 3b, 4 ± 3 vs. 8 ± 3 ml, CPB + AP vs. CPB, *p* = 0.005).

### Renal endothelial architecture, neutrophil infiltration and fibrin deposition

**Fig. 4:**
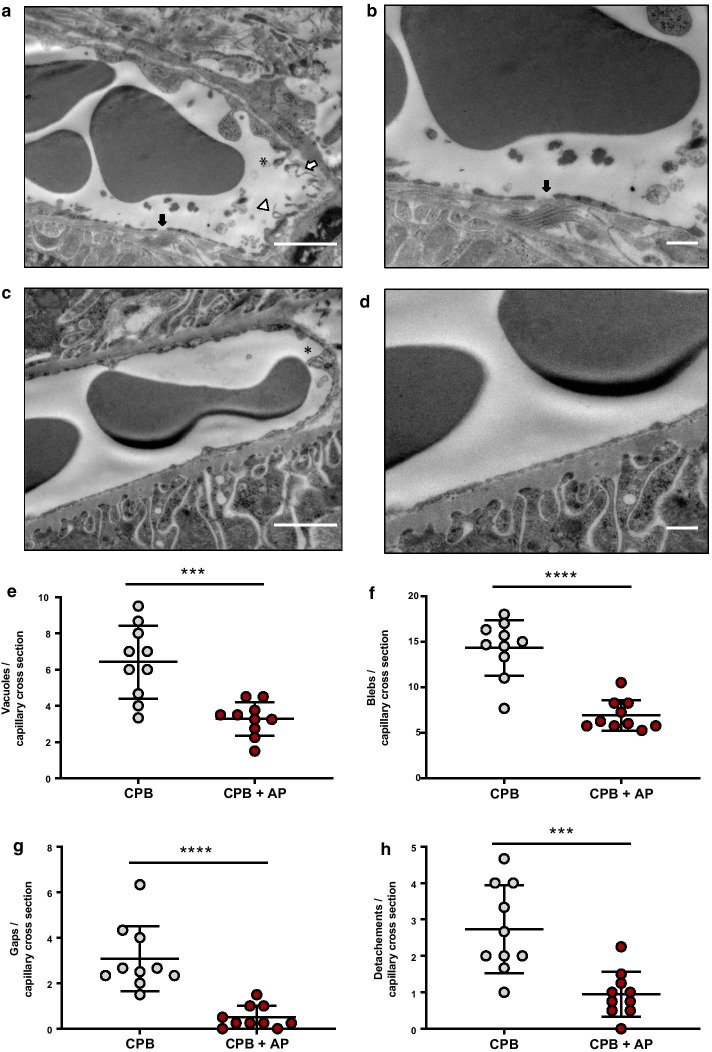
Renal endothelial structure. Electron microscopy images of kidney tissue of rats undergoing cardiopulmonary bypass (**a**, **b**, CPB, *n* = 3, with different magnification) and CPB with aprotinin treatment (**c**, **d**, CPB + AP, *n* = 4, with different magnification) and quantification of endothelial vacuolization (white triangle, **e**), membrane blebbling (black star, **f**), renal endothelial gap formation (black arrow, **G**) and endothelial cell detachment from basement membrane (white arrow, **h**). Data are presented as mean ± standard deviation, ***p < 0.0005, ****p < 0.0001 CPB vs. CPB + AP

Renal endothelial ultrastructure was better preserved in rats treated with aprotinin compared to untreated animals (Fig. 4). Aprotinin reduced endothelial vacuolization (Fig. 4e, 3.3 ± 0.9 vs. 6.4 ± 2.0 vacuoles/capillary cross section, CPB + AP vs. CPB, *p* = 0.0005) and membrane blebbing (Fig. 4f, 6.9 ± 1.7 vs. 14.3 ± 3.1 blebs per capillary cross section, CPB + AP vs. CPB, *p* < 0.0001). Moreover, aprotinin treatment reduced renal endothelial gap formation (Fig. 4g, 0.5 ± 0.5 vs. 3.1 ± 1.4 gaps per capillary cross section, CPB + AP vs. CPB, *p* < 0.0001) and endothelial cell detachment from basement membrane (Fig. 4h, 1.0 ± 0.6 vs. 2.7 ± 1.2 detachments per capillary cross section, CPB + AP vs. CPB, *p* = 0.0006).

**Fig. 5:**
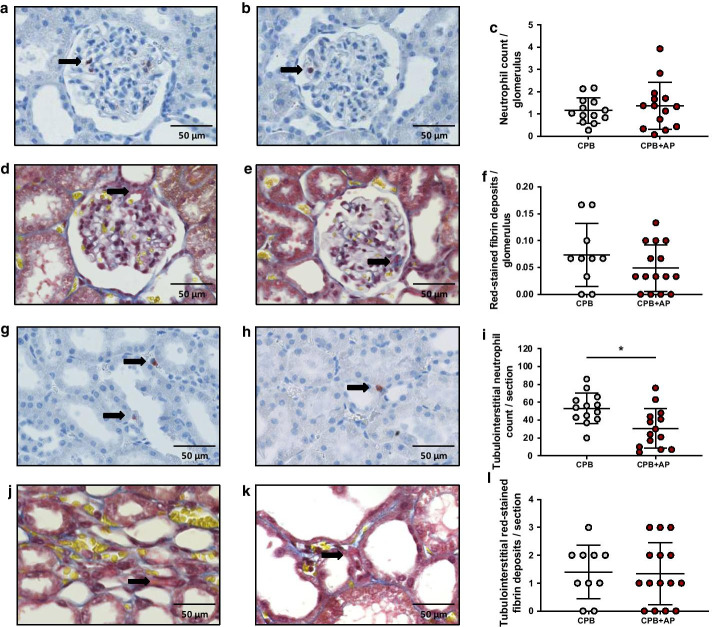
Glomerular and tubulointerstitial neutrophil infiltration and fibrin deposition. Glomerular neutrophil infiltration in rats undergoing cardiopulmonary bypass (**a**, CPB; grey circles, *n* = 13) and CPB with aprotinin treatment (**b** CPB + AP, red circles, *n* = 14) and quantified as MPO-positive cells per glomerulus (**c**). Glomerular fibrin deposition in rats undergoing CPB (**d**, grey circles, *n* = 10) and CPB with aprotinin treatment (**e**, red circles, *n* = 15) and quantified as red stained MSB deposits per glomerulus (**f**). Black arrows show representative stainings. Tubulointerstitial neutrophil infiltration in rats undergoing cardiopulmonary bypass (**g**, CPB; grey circles, *n* = 13) and CPB with aprotinin treatment (**h** CPB + AP, red circles, *n* = 14) and quantified as MPO-positive cells per glomerulus (**i**). Glomerular fibrin deposition in rats undergoing CPB (**j**, grey circles, *n* = 10) and CPB with aprotinin treatment (**k**, red circles, *n* = 15) and quantified as red stained MSB deposits per glomerulus (**l**). Black arrows show representative stainings. Data are presented as mean ± standard deviation, *p < 0.001 CPB vs. CPB + AP

Moreover, aprotinin treatment reduced tubulointerstitial neutrophil influx by 1.7-fold compared to untreated CPB rats (Fig. 4g–i, 30.7 ± 22.1 vs. 53.2 ± 17.2 tubulointerstitial neutrophil influx per section, CPB + AP vs. CPB, *p* = 0.009). Aprotinin treatment did not affect glomerular neutrophil infiltration (Fig. 5a–c, 1.4 ± 1.1 vs. 1.2 ± 0.6 neutrophil influx per glomerulus, CPB + AP vs. CPB, *p* = 0.73), fibrin-stained glomerular thrombi (Fig. 5d–f, 0.049 ± 0.043 vs. 0.073 ± 0.058 MSB + foci per glomerulus, CPB + AP vs. CPB, *p* = 0.33), nor fibrin-stained tubulointerstitial thrombi (Fig. 5j–l, 1.3 ± 1.1 vs. 1.4 ± 1.0 MSB + foci per section, CPB + AP vs. CPB, *p* = 0.8) compared to untreated CPB rats.

### Coagulation and inflammatory signaling

CPB increased circulating levels of IL-6, vWF, sTM and TAT, which further increased in the first hour after weaning from CPB (Table 1). Aprotinin did not affect circulating levels of these molecules at baseline nor during the course of the experiment (Table 1). Moreover, no differences in renal adhesion molecule expression (ICAM-1, VCAM-1, E-selectin and P-selectin gene expression) was observed one hour after weaning from CPB between groups (Table 1). Also, no differences in PAR1 gene nor protein levels were observed between groups (Table 1).

**Table 1:** Plasma and urine concentrations and renal protein and gene expression levels of inflammatory, coagulation, and renal injury molecules

		Baseline		60 min during CPB		60 min post-CPB	
		CPB control	CPB Aprotinin	CPB control	CPB Aprotinin	CPB control	CPB Aprotinin
Plasma	IL-6 (pg/ml)	21 ± 3	16 ± 9	71 ± 16*	85 ± 32*	772 ± 354*	775 ± 235*
	vWF (ng/ml)	159 ± 40	163 ± 23	243 ± 32*	240 ± 57*	275 ± 98*	248 ± 54*
	sTM (ng/ml)	0.2 ± 0.2	0.2 ± 0.2	1.0 ± 0.6*	1.1 ± 0.6*	6.0 ± 0.7*	6.0 ± 1.5*
	TAT (ng/ml)	1.6 ± 0.8	1.7 ± 1.0	1.5 ± 0.7	1.6 ± 0.6	2.9 ± 1.4*	2.9 ± 1.0*
	NGAL (ng/ml)	48 ± 7	43 ± 9	–	–	2012 ± 970*	1880 ± 700*
	KIM-1 (pg/ml)	177 ± 27	193 ± 31	–	–	273 ± 81*	239 ± 48
	Creatinine (nmol/ml)	23 ± 1	24 ± 1	–	–	58 ± 12*	57 ± 10*
Urine	NGAL (ng/ml)	–	–	–	–	659 ± 676	524 ± 387
	KIM-1 (pg/ml)	–	–	–	–	1281 ± 865	1103 ± 703
Gene expression	ICAM-1	–	–	–	–	71 ± 56	68 ± 48
	VCAM-1	–	–	–	–	1.8 ± 1.1	2.0 ± 0.8
	E-selectin	–	–	–	–	0.09 ± 0.04	0.10 ± 0.05
	P-selectin	–	–	–	–	0.11 ± 0.07	0.10 ± 0.04
	PAR1	–	–	–	–	0.63 ± 0.30	0.78 ± 0.23
	NGAL	–	–	–	–	29 ± 17	31 ± 16
	KIM-1	–	–	–	–	9 ± 8	7 ± 6
Protein expression	PAR1	–	–	–	–	9.5 ± 6.7	10.0 ± 4.6

Data are presented as mean ± standard deviation

*CPB* cardiopulmonary bypass, *IL-6* interleukin-6, *ICAM-1* intracellular adhesion molecule 1, *VCAM-1* vascular cell adhesion molecule 1, *vWF* von Willebrand Factor, *sTM* soluble thrombomodulin, *TAT* thrombin–antithrombin complex, *PAR1* protease-activated receptor 1, *NGAL* neutrophil gelatinase-associated lipocalin, *KIM-1* kidney injury molecule-1

^*^p < 0.05 versus baseline, #*p* < 0.05 CPB control versus CPB aprotinin

### Renal injury

CPB was associated with increased plasma and urine concentrations of renal injury markers NGAL, KIM-1 and creatinine one hour after weaning from CPB (Table 1). Aprotinin did not affect plasma or urine concentrations of renal injury markers nor renal injury gene expression levels (Table 1). The elevation of plasma renal injury markers was observed in both CPB groups 60 min post-CPB, however, not in sham animals (Additional file 2: Figure S3).

## Discussion

In the present study, we demonstrate that aprotinin administration preserved renal endothelial structure and reduced renal edema formation following experimental CPB. This was associated with reduced fluid resuscitation requirements and restoration of cremaster microcirculatory perfusion. These findings are important as they emphasize on the broad spectrum of actions of aprotinin. Besides prevention of blood loss and preservation of platelet function, we showed that aprotinin preserved renal endothelial ultrastructure following CPB. Despite its endothelial protective effect, aprotinin administration was however not sufficient to preserve renal perfusion nor reduce renal injury markers the first hour after weaning from CPB.

Previously, we showed that plasma from patients undergoing cardiac surgery with CPB resulted in loss of in vitro endothelial barrier function [28]. This plasma-induced endothelial hyperpermeability continued in the first postoperative days and correlated with in vivo microcirculatory perfusion disturbances [29]. These results emphasize capillary leakage as underlying mechanism of CPB-induced microcirculatory perfusion disturbances and AKI. Additionally, supporting this hypothesis, we previously showed that therapeutic inhibition of capillary leakage by imatinib and vasculotide, drugs that improve vascular barrier function, improved cremaster microcirculatory perfusion and reduced organ edema following experimental CPB [14, 15]. Despite these promising findings, vasculotide was effective in reducing only pulmonary edema but not renal edema. In contrast, imatinib was found to reduce renal edema and markers of renal injury, however, its use is associated with a wide range of side effects and is registered for the treatment of chronic myeloid leukemia, which may hamper its implementation in cardio-surgical setting.

Aprotinin is suggested to reduce capillary leakage and is of interest as it is already used in cardiac surgery to inhibit hyperfibrinolysis. In patients undergoing cardiac surgery, aprotinin is thought to decrease pulmonary edema [30]. In experimental CPB, aprotinin reduced myocardial edema and fluid requirements [31]. Our data confirm these results as we demonstrated that aprotinin administration maintained renal endothelial structure and prevented renal edema formation. Additionally, less fluids were required following aprotinin administration to maintain adequate pump flow. These results suggest that aprotinin has endothelial barrier protective effects resulting in less capillary leakage and edema formation in kidneys during experimental CPB.

As a non-specific serine protease inhibitor, aprotinin operates via numerous mechanisms [32]. Aprotinin can prevent thrombin from cleaving and activating PAR1 [21–24]. Thrombin regulates endothelial permeability, vascular tone, leukocyte trafficking and inflammation, formation of new vessels, and hemostasis. PAR1 is the principal receptor for thrombin and is shown to regulate endothelial permeability. In the present study, onset of CPB resulted in thrombin generation as assessed by thrombin–antithrombin complex formation. However, we did not find an effect of aprotinin on renal PAR1 expression. This might be due to the lack of available antibodies to assess the activation status of PAR1. As a consequence, only uncleaved PAR1 could be determined. However, we cannot rule out that aprotinin activated alternative mechanisms preserving endothelial barrier function, as it has been implicated that aprotinin inhibits plasma leakage induced by kallikrein. Moreover, aprotinin has anti-inflammatory properties. In previous studies, aprotinin reduced thrombin-induced IL-6 secretion [23] and TNFα-induced ICAM-1 and VCAM-1 expression [21–24] by endothelial cells in vitro. In addition, aprotinin inhibited leukocyte extravasation in rats [33]. In line, we found reduced tubulointerstitial neutrophil extravasation in aprotinin-treated animals compared to control animals. Interestingly however, circulating inflammatory mediators were comparable between aprotinin-treated and untreated rats. This contrasting finding could result from dose-dependent actions of aprotinin. Aprotinin seems to be more effective at high doses and when given during reperfusion [33], which may potentially partly be explanatory for the lack of preservation of renal perfusion and function in our study. Collectively, aprotinin seems to target multiple mechanisms and the effects on the endothelium remain complex and need further investigation.

As a consequence of capillary leakage, we hypothesized the development of microcirculatory perfusion disturbances and AKI [14, 15]. Patients undergoing cardiac surgery with CPB show sublingual microcirculatory perfusion disturbances [7, 8] and it is suggested that these microcirculatory perfusion disturbances play a crucial role in the development of AKI [34, 35]. The question remains whether sublingual measurements can be translated to other organs, such as kidneys. The present study showed impaired renal perfusion following CPB which was indeed associated with by increased markers of renal injury. As aprotinin was not sufficient to preserve renal perfusion, the hypothesized causality between renal perfusion disturbances and renal injury following CPB could not be confirmed. Interestingly, the present study demonstrates that aprotinin administration restored cremaster microcirculatory, but not renal perfusion. Previously, these differences between microvascular beds was observed by our group in a cell-type specific effect of plasma-induced endothelial hyperpermeability, which was more severe in pulmonary endothelial cells compared to renal endothelial cells [29]. In line with these findings, we observed that aprotinin more effectively reduced pulmonary edema compared to renal edema (data not shown). Another explanation is that cremaster perfusion was specifically measured in the capillaries, while renal perfusion analysis included vessels of all sizes. The effects of aprotinin on renal perfusion are however scarcely studied. In patients undergoing cardiac surgery with CPB, renal plasma flow decreased following CPB and was not affected by aprotinin administration [36, 37]. Even though the effects of aprotinin on renal perfusion during CPB requires further investigation, these data implicate organ specific effects of aprotinin.

Aprotinin was reintroduced in 2017, 10 years after it was taken of the market. Conflicting results have been reported concerning the effect of aprotinin during cardiac surgery on postoperative morbidity, including renal function. [38, 39] However, validity was questioned, as most studies that reported increased postoperative morbidity risk following aprotinin administration were subjected to sources of bias and were not designed to look at safety. When reviewing randomized placebo-controlled trials, aprotinin use during cardiac surgery was not associated with increased risk of postoperative morbidity, including renal injury, or mortality [40]. In accordance, we did not observe deleterious effects of aprotinin administration on renal function in our rat CPB model.

Controversy exists concerning the effect of CPB on the development of AKI. Large randomized trials revealed no differences on occurrence of postoperative AKI between off-pump and on-pump coronary artery bypass grafting, indicating that in particular non-CPB factors may contribute to postoperative AKI [41, 42]. However, these findings should be placed into perspective as most of these trials were subjected to imbalanced exclusion of patients assigned to off-pump surgery and primarily focused on mortality rates and renal replacement therapy as outcome measures. Renal replacement therapy as outcome is not representative for the prevalence of CPB-associated renal injury, as the incidence of AKI requiring dialysis is usually low (up to 4%), whereas less severe forms of postoperative renal dysfunction, while still being clinically relevant, are observed in 5 to 53% of the patients [43–45]. When interpreting these trials, it is important to consider that the incidence of AKI depends on studied cardiac surgical population, preoperative renal function status, and criteria used to define acute kidney injury. In addition, magnitude of tubular injury and occurrence of postoperative AKI appears independently associated with duration of CPB, duration of low oxygen delivery (DO_2_) during CPB, and degree of rewarming [46, 47].

This study has limitations. We choose to study a clinically relevant dose (reflecting a full Hammersmith protocol) based on previous studies that showed that patients receiving full Hammersmith aprotinin protocol showed significant reductions in circulating interleukin levels. However, in our study, aprotinin was not continuously infused nor added to CPB prime solution and may therefore have not been sufficient to suppress the systemic inflammatory response, potentially the consequence of first pass effect. Nevertheless, based on our observation in the cremaster muscle, a biological effect of aprotinin was clearly present. Moreover, a potential beneficial effect of aprotinin administration on renal function may have been masked by the decrease in blood pressure following CPB and the relatively short follow-up period following weaning from CPB. Also, our extracorporeal circuit was primed with hydroxyethyl starch, a colloid solution. Although concerns have been raised concerning the effect of colloids on renal function, the use of modern hydroxyethyl starch solutions in surgical settings, including cardiac surgery, was not associated with increased risk of acute kidney injury [40]. Moreover, when interpreting our data, one should take into consideration that cardiac surgery associated renal injury is multifactorial. Within our experimental set-up, we focused primarily on CPB-associated renal perfusion disturbances as main contributor to AKI. Aside from renal perfusion disturbances, also CPB-induced hemolysis through mechanical destruction of erythrocytes caused by contact of blood with the bypass circuit and cardiotomy suction, was not taken into account [48]. This was partly avoided, as sternotomy and cardiotomy suction were not performed in our experimental CPB model. Moreover, post-CPB concentrations of NGAL did not differ between studied CPB groups, which is suggested to correlate with both AKI and hemolysis in the clinical setting [49]. However, although sternotomy and cardioplegic arrest were avoided, our CPB model did enable discontinuation of ventilation and ensures systemic oxygenation while minimizing cardiac blood flow and pulse pressure, thereby mimicking true CPB effect. This is in accordance with our findings that an additional group of sham rats, that received similar anesthetic and surgical procedures, did not show described perfusion disturbances and elevation of renal injury markers during our experimental study period (Additional file 2).

## Conclusions

CPB impairs renal endothelial integrity and is associated with renal edema and renal microcirculatory perfusion disturbances. Our study revealed that although aprotinin protects renal endothelial integrity and reduced renal edema, this strategy was not sufficient to preserve renal perfusion nor reduce renal injury during the first hour following CPB. The findings of our study highlight the importance of the development of new therapeutic interventions to improve renal perfusion, particularly in the setting of cardiac surgery with CPB.

## Supplementary Information

**Additional file 1:** Additional methods.

**Additional file 2:  Figure S1.** Mean arterial pressure (B) and hematocrit levels (C) in rats during and following cardiopulmonary bypass (CPB; white boxes; *n*=15), CPB with aprotinin treatment (CPB+AP; red boxes; *n*=15), or sham rats (Sham; grey boxes *n*=9). Boxes and whiskers represent median, interquartile and full range, * p < 0.05 CPB vs. CPB baseline, # p < 0.05 CPB groups vs. sham. **Figure S2.** Renal blood volume (A), renal vascular filling velocity (B), and estimate of renal perfusion (C) measured in the right renal cortex using contrast enhanced ultrasound in sham rats (Sham; grey boxes; *n*=9), rats undergoing cardiopulmonary bypass (CPB; white boxes; *n*=15) or rats undergoing CPB with aprotinin treatment (CPB+AP; red boxes; *n*=15). Boxes and whiskers represent median, interquartile and full range, * p < 0.05 CPB vs. CPB baseline, # p < 0.05 CPB vs. sham. **Figure S3.** Plasma concentrations of renal injury molecules NGAL (A), KIM-1 (B), and creatinine (C) measured 1 hour (1h) after weaning from CPB. Data are presented as mean ± standard deviation. A black bar represents p < 0.05 CPB vs sham. CPB, cardiopulmonary bypass; NGAL, neutrophil gelatinase-associated lipocalin; KIM-1, kidney injury molecule-1.

## Data Availability

The datasets used and/or analyzed during the current study are available from the corresponding author on reasonable request.

## Abbreviations

AKI: Acute kidney injury
AP: Aprotinin
CPB: Cardiopulmonary bypass
ELISA: Enzyme-linked immunosorbent assay
ICAM-1: Intercellular adhesion molecule 1
IL-6: Interleukin-6
Kg: Kilogram
KIM-1: Kidney injury molecule-1
Min: Minutes
MPO: Myeloperoxidase
MSB: Martius, Scarlet and Blue
NGAL: Neutrophil gelatinase-associated lipocalin
PAR1: Protease-activated receptor 1
PBS: Phosphate buffered solution
Sec: Second(s)
sTM: Soluble thrombomodulin
TAT: Thrombin–antithrombin complex
TNFα: Tumor necrosis factor alpha
VCAM-1: Vascular cell adhesion molecule 1
vWF: Von Willebrand Factor
