# Supplementary material for: Preservation of renal endothelial integrity and reduction of renal edema by aprotinin does not preserve renal perfusion and function following experimental cardiopulmonary bypass
Source: Intensive Care Med Exp. 2021 Jun 25;9:30. doi: 10.1186/s40635-021-00393-9 (PMC8225734; doi:10.1186/s40635-021-00393-9)
Supplement: Supplementary file 1 — Additional file 1: Additional methods. [file 40635_2021_393_MOESM1_ESM.docx]

**Preservation of renal endothelial integrity and reduction of renal edema by aprotinin does not preserve renal perfusion and function following experimental cardiopulmonary bypass**

Nicole A.M. Dekker, M.D.^1,2,3,+^, Anoek L.I. van Leeuwen. B.Sc.^1,2,3^, Matijs van Meurs, M.D., Ph.D.^4,5^, Jill Moser, Ph.D.^4,5^, Jeannette E. Pankras, B.sc^6^, Nicole N. van der Wel, Ph.D^6^, Hans W. Niessen, Ph.D.^7^, Marc G. Vervloet, M.D., Ph.D.,^8^Alexander B.A. Vonk, M.D., Ph.D.^2^, Peter L. Hordijk, Ph.D.^3^, Christa Boer, Ph.D.^1^, Charissa E. van den Brom, Ph.D.^1,3,9^

**SUPPLEMENTAL METHODS**

*Animals*

Male Wistar rats of 375-425 grams (Charles River Laboratories, Brussels, Belgium) were housed in a temperature-controlled room (12/12 hours light dark cycle, 20–23°C, 40-60% humidity) with food and water *ad libitum*.

Male Wistar rats of 375-425 gram (Charles River Laboratories, Brussels, Belgium) were randomly assigned to undergo cardiopulmonary bypass (CPB) with aprotinin treatment (CPB+AP, *n*=15) or phosphate buffered solution (PBS) as control (CPB, *n*=15) for measurement of cremaster and renal perfusion (Figure 1A). An additional group of sham rats (SHAM *n*=9) were included to asses CPB-related effect on renal perfusion and plasma renal injury markers (supplemental data file).

Rats were culled by blood withdrawal under 5.0% isoflurane inhalation 60 minutes after weaning from CPB. Blood was protracted from the femoral arterial line in approximately 1 minute using a 10 mL syringe. Kidneys were isolated and blood and urine samples were collected and stored at -80°C for additional molecular analyses.

Surgeries were performed under isoflurane, fentanyl and local lidocaine anesthesia and analgesia, and all efforts were made to minimize animal suffering. Depth of anaesthesia was continuously monitored and adjusted if necessary based on heart rate and mean arterial pressure. Moreover, adequate depth of anaesthesia was confirmed by the absence of the hind limb withdrawal reflex.

*Anesthesia and surgical preparation*

All animals were anesthetized as previously reported^14,15^ with 4.0% isoflurane (Ivax Farma, Haarlem, The Netherlands) in oxygen. Following endotracheal intubation with a 16G catheter (Venflon Pro, Becton Dickinson, Helsingborg, Sweden), lungs were mechanically ventilated (UMV-03, UNO Roestvaststaal BV, Zevenaar, The Netherlands; PEEP 2-4 cm H_2_O, respiratory rate of 60-65 breaths/min, tidal volume ~10 ml/kg) and anesthesia was maintained with 1.5-2.0% isoflurane in oxygen-enriched air (40% O_2_/ 60% N_2_). Additionally, fentanyl boluses (12µg/kg) were administered every 30-40 minutes. Respiratory rate was adjusted based on blood gas values to maintain pH and partial pressure of carbon dioxide within physiological limits. Depth of anaesthesia was continuously monitored and adjusted if necessary based on heart rate and mean arterial pressure. Body temperature was continuously measured and maintained stable between 36.5 and 37.5°C using a temperature controller (TC-1000 Rat, CWe Inc., United States). A 22G catheter (Venflon Pro, Becton Dickinson, Helsingborg, Sweden) was placed in the caudal (tail) artery for continuous measurements of arterial blood pressure and blood withdrawal for blood gas analysis and hematocrit measurements (ABL80, radiometer, Copenhagen, Denmark). Fentanyl 12 µg/kg (Janssen-Cilag, Tilburg, The Netherlands) was administered as additional analgesia and repeated approximately every 30-40 minutes during the experimental procedure. Arterial blood gas and hematocrit measurements were performed at baseline and repeated at 10, 30, and 60 min of extracorporeal circulation and 10 and 60 min after weaning from CPB. Arterial blood pressure, ECG and heart rate were continuously recorded using PowerLab software (PowerLab 8/35, Chart 8.0; AD Instruments Pty, Ltd., Castle Hill, Australia).

The left cremaster muscle was isolated and prepared for cremaster perfusion measurements as previously described.^14,15^ Heparin (500 IU/kg, LEOPharma, Amsterdam, The Netherlands) was administered followed by cannulation of the right femoral artery with a 20G catheter (Arterial Cannula, Becton Dickinson, Helsingborg, Sweden) for arterial inflow of the CPB circuit. The right jugular vein was catheterized with a 20G catheter (Venflon Pro, Becton Dickinson, Helsingborg, Sweden) for administration of aprotinin or PBS, and the administration of microbubbles for contrast enhanced echography (CEUS).

*Cardiopulmonary bypass*

The protocol for CPB was performed as previously described.^14,15^ In summary, the CPB circuit consisted of an open venous reservoir, a roller pump (Pericor SF70, Verder, Haan, Germany), and an oxygenator-heat exchanger with a three-layer hollow fiber membrane for gas exchange (Ing. M. Humbs, Valley, Germany) (Figure 1). A 1.0-mm-diameter arterial line (LectroCath, Vygon, Ecouen, France) was connected to the femoral inflow catheter. The circuit was primed with 10 ml of 6% hydroxyethyl starch (HES; Voluven, Fresenius Krabi, Halden, Norway). The right jugular vein catheter was replaced by a modified multiorifice 4.5 French catheter (Desilets-Hoffman, Cook, Bloomington, IN) that was introduced into the right atrium, followed by initiation of CPB. After onset of CPB, ventilation was discontinued and a mixture of oxygen, carbon dioxide and isoflurane (0.8-1.5%) was led through the oxygenator membrane of the extracorporeal circuit. During extracorporeal circulation, temperature was maintained between 35.0 and 35.5°C. Additional doses of 6% hydroxyethyl starch (1 mL bolus; 6%HES; Voluven, Fresenius Krabi, Halden, Norway) were administrated when necessary to maintain target CPB flow rates >150 ml/kg/min. Hematocrit levels were checked to avoid excessive fluid resuscitation. At 65 min of CPB, ventilation was restarted at a frequency of 30 breaths/min and rats were rewarmed to 36.5°C. After increasing the respiratory rate to 60-65 breaths/min, rats were weaned from CPB at 75 min of extracorporeal circulation. Fifteen minutes after weaning from CPB, protamine hydrochloride (2 mg/kg) was administered to neutralize heparin.

*Cremaster microcirculatory perfusion*

After stabilisation of the exposed cremaster muscle for at least 30 minutes, microcirculatory perfusion measurements were performed using a 10x objective on an intravital microscope (AxiotechVario 100HD, Zeiss, Oberkochen, Germany) connected to a digital camera (scA640, Basler, Ahrensburg, Germany) with a final magnification of 640x as described previously.^14,15^ Briefly, three regions of the microvasculature in the cremaster muscle with adequate perfusion quality were selected during baseline. These exact predefined regions were followed throughout the experiment. Measurements were performed directly after the surgical preparation (baseline), 10 minutes after initiation of CPB (10’ CPB), 60 min after start of CPB (60’ CPB), 10 min after weaning of CPB (10’ post-CPB) and 60 min after weaning of CPB (60’ post-CPB), see also Figure 1A.

Microcirculatory perfusion analyses were performed offline by an investigator who was blinded for treatment allocation. To objectify microcirculatory perfusion, each video screen was divided into three parts by two vertical lines.^14 15^ The total number of capillaries per screen was obtained by averaging the counted capillary crossings per part of the screen. These small vessels were categorised in continuously perfused (continuous blood flow), intermittently perfused (blood flow was arrested at least once or flow was reversed) and non-perfused capillaries (no flow). Eventually, the proportion of continuously perfused vessels was calculated by the ratio of the absolute number of continuously perfused capillaries (averaged for each time point) and the total number of capillaries. Similar calculations were performed for intermittently perfused and non-perfused vessels.

*Renal perfusion*

Contrast enhanced echography was performed as previous described^26^ with a Vevo 2100 Imaging System and MS 250 Nonlinear Contrast Imaging transducer (VisualSonics Inc, Toronto, Canada). Microbubbles were continuously infused via the jugular vein with a rate of 150 µl/min and the right kidney was visualized in the longitudinal plane at predefined measurement time points (Figure 1). After two minutes of microbubble infusion at low acoustic power until steady state was reached, a burst of high acoustic power was applied to destroy the microbubbles. Subsequently, images during low acoustic power were acquired to allow bubble-contrast replenishment in the kidney. This destruction-replenishment sequence was repeated two times.

For each measurement, regions of interest were drawn in the renal cortex by an investigator who was blinded for treatment allocation. Renal signal intensities from the frames after microbubble destruction were corrected for background noise by subtracting the signal intensity of the first frame after microbubble destruction (𝑌0). These intensities were then fitted (𝑌 = 𝑌0+ (𝐴 − 𝑌0) ⋅ (1 − exp^(−𝛽⋅𝑥)^)) for calculation of renal vascular blood volume (𝐴) and renal vascular filling velocity (𝛽), which corresponds to renal blood exchange rate. The estimate of renal perfusion was calculated as the product of 𝐴 and 𝛽.

*Electron microscopy*

Renal tissue (*n*=3-4 per group) was used for analysis of renal cortical capillary endothelial ultrastructure.^14^ Renal tissue was harvested at the end of the experiment and immediately fixed in Karnovsky fixative. Subsequently, tissue was post fixed in osmium tetroxide 1% and dehydrated through a graded series of ethanol solutions of 70-100% and impregnated with propylene oxid 1:1 and embedded in L-112 Resin. Thereafter, ultra-thin (50-70 nm) sections were cut with a diamond knife. The sections were contrasted with uranyl acetate and lead citrate. Ten random capillaries in the renal cortex were captured using electron microscope imaging (Tecnai 12G^2^, Fei company, with a VALETA side entry camera) by an investigator blinded to group allocation. Subsequently, images were analyzed for signs of endothelial injury and activation, including luminal membrane blebs, vacuoles, endothelial gaps and endothelial detachment.

*Plasma and urinary analyses*

Arterial blood was collected in EDTA tubes at baseline, 60 minutes after initiation of CPB and one hour after weaning from CPB. Blood was centrifuged twice to obtain platelet free plasma and stored at -80°C. Urine samples were obtained one hour after weaning of CPB (60 min post-CPB). Urinary levels of neutrophil gelatinase-associated lipocalin (NGAL) and kidney injury molecule-1 (KIM-1) and plasma levels of NGAL, KIM-1, creatinine, thrombin-antithrombin complex (TAT), soluble thrombomodulin (sTM), von Willebrand Factor (vWF) and interleukin-6 (IL-6) were measured with ELISA (Cloud-Clone Corporation, Wuhan, Hubei, China) in accordance to the manufacturer.

*Immunohistochemical analyses*

Renal tissue was fixed in 4% formalin and embedded in paraffin. Longitudinal sections of 4 µm were cut and mounted on 3-aminopropyltriethoxisilane-coated slides (Superfrost,® Plus, Menzal, Darmstadt, Germany). After deparaffinisation and rehydration, immunohistochemical staining was performed for assessment of glomerular and tubulointerstitial neutrophil infiltration by anti-myeloperoxidase (MPO; 1:50, Abcam, Cambridge, UK)^14^ and for assessment of fibrin-stained thrombi deposition by Martius, Scarlet and Blue (MSB) staining. In anti-MPO stained sections, three regions were selected and the number of neutrophils were manually counted in 10 glomeruli per region and total count of neutrophils within the tubulointerstitial space per section (Aperio ImageScope x64, Aperio Technologies). In MSB stained sections, the number of red-stained MSB positive foci in glomeruli were manually counted and divided by the total count of glomeruli.^27^ For quantification of tubulointerstitial fibrin deposits, the total number of red-stained MSB foci within the tubulointerstitial space were counted per section. The investigator performing the analysis was blinded to group allocation.

*Gene expression*

Total RNA was extracted from 10-30 mg frozen kidney tissue and isolated using the RNeasy mini kit (Qiagen, Venlo, The Netherlands), as previously described.^15^ The following genes were used for quantitative PCR: KIM-1, NGAL, ICAM-1, VCAM-1, E-selectin, P-selectin and PAR1 (Applied Biosystems, Foster City, CA). ΔcT was calculated and mRNA expression levels were normalized to the housekeeping gene Tfrc. Values outside of the measurement detection range were excluded for analysis.

*Protein expression*

Frozen kidney tissue was homogenized to obtain cellular protein fractions for western blot analysis as described previously.^15,26^ Protein expression of uncleaved PAR1 was analyzed using anti-PAR1/Thrombin Receptor (25 µg protein, 1:200, ab32611, Abcam, USA). Immunoblots were quantified by densitometric analysis of films (ImageQuant TL, v8.1, GE Healthcare, USA). Signal was normalized to glyceraldehyde 3-phosphate dehydrogenase protein expression (GAPDH; 1:5000, No. 2118, Cell Signalling Technology, USA).
