# Supplementary material for: Preservation of renal endothelial integrity and reduction of renal edema by aprotinin does not preserve renal perfusion and function following experimental cardiopulmonary bypass
Source: Intensive Care Med Exp. 2021 Jun 25;9:30. doi: 10.1186/s40635-021-00393-9 (PMC8225734; doi:10.1186/s40635-021-00393-9)
Supplement: Supplementary file 2 — Additional file 2: Figure S1. Mean arterial pressure (B) and hematocrit levels (C) in rats during and following cardiopulmonary bypass (CPB; white boxes; n=15), CPB with aprotinin treatment (CPB+AP; red boxes; n=15), or sham rats (Sham; grey boxes n=9). Boxes and whiskers represent median, interquartile and full range, * p < 0.05 CPB vs. CPB baseline, # p < 0.05 CPB groups vs. sham. Figure S2. Renal blood volume (A), renal vascular filling velocity (B), and estimate of renal perfusion (C) measured in the right renal cortex using contrast enhanced ultrasound in sham rats (Sham; grey boxes; n=9), rats undergoing cardiopulmonary bypass (CPB; white boxes; n=15) or rats undergoing CPB with aprotinin treatment (CPB+AP; red boxes; n=15). Boxes and whiskers represent median, interquartile and full range, * p < 0.05 CPB vs. CPB baseline, # p < 0.05 CPB vs. sham. Figure S3. Plasma concentrations of renal injury molecules NGAL (A), KIM-1 (B), and creatinine (C) measured 1 hour (1h) after weaning from CPB. Data are presented as mean ± standard deviation. A black bar represents p < 0.05 CPB vs sham. CPB, cardiopulmonary bypass; NGAL, neutrophil gelatinase-associated lipocalin; KIM-1, kidney injury molecule-1. [file 40635_2021_393_MOESM2_ESM.docx]

**Preservation of renal endothelial integrity and reduction of renal edema by aprotinin does not preserve renal perfusion and function following experimental cardiopulmonary bypass**

Nicole A.M. Dekker, M.D.^1,2,3,+^, Anoek L.I. van Leeuwen. B.Sc.^1,2,3^, Matijs van Meurs, M.D., Ph.D.^4,5^, Jill Moser, Ph.D.^4,5^, Jeannette E. Pankras, B.sc^6^, Nicole N. van der Wel, Ph.D^6^, Hans W. Niessen, Ph.D.^7^, Marc G. Vervloet, M.D., Ph.D.,^8^Alexander B.A. Vonk, M.D., Ph.D.^2^, Peter L. Hordijk, Ph.D.^3^, Christa Boer, Ph.D.^1^, Charissa E. van den Brom, Ph.D.^1,3,9^

**Additional Data**

*Sham animals*

An additional group of sham rats (SHAM *n*=9) were included to asses CPB-related effect on renal perfusion and plasma renal injury markers. The sham procedure was identical to the CPB groups except for initiation and weaning of CPB, but including heparin, rocuronium bromide and protamine administration. No other interventions were made throughout the procedure, and all analyses occurred identical as in the CPB group protocols. Arterial blood gas and hematocrit measurements were performed at baseline and repeated at corresponding time points of CPB groups for sham experiments.


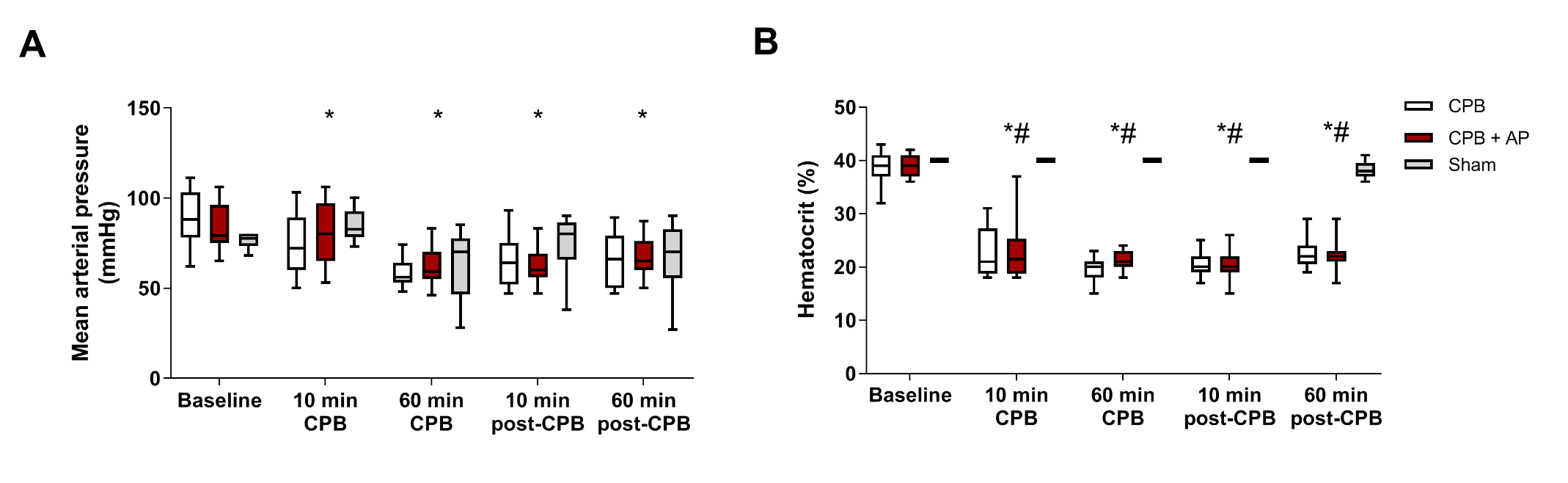


**Figure S1.** Mean arterial pressure (**B**) and hematocrit levels (**C**) in rats during and following cardiopulmonary bypass (CPB; white boxes; *n*=15), CPB with aprotinin treatment (CPB+AP; red boxes; *n*=15), or sham rats (Sham; grey boxes *n*=9). Boxes and whiskers represent median, interquartile and full range, * p < 0.05 CPB vs. CPB baseline, # p < 0.05 CPB groups vs. sham.

*
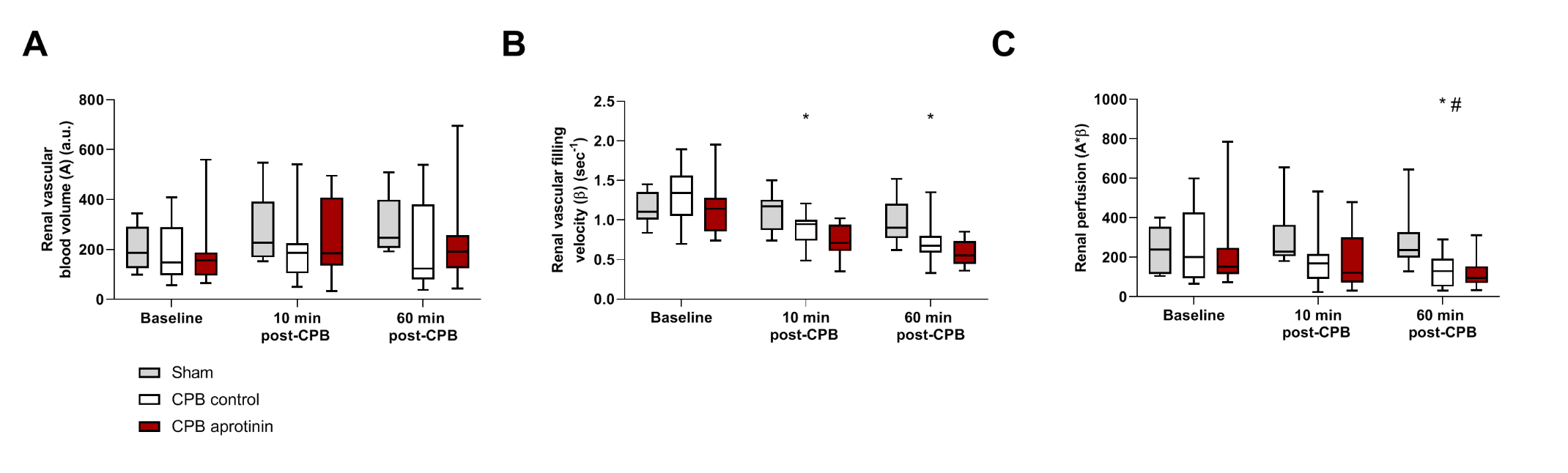
*

**Figure S2.** Renal blood volume (**A**), renal vascular filling velocity (**B**), and estimate of renal perfusion (**C**) measured in the right renal cortex using contrast enhanced ultrasound in sham rats (Sham; grey boxes; *n*=9), rats undergoing cardiopulmonary bypass (CPB; white boxes; *n*=15) or rats undergoing CPB with aprotinin treatment (CPB+AP; red boxes; *n*=15). Boxes and whiskers represent median, interquartile and full range, * p < 0.05 CPB vs. CPB baseline, # p < 0.05 CPB vs. sham.

*
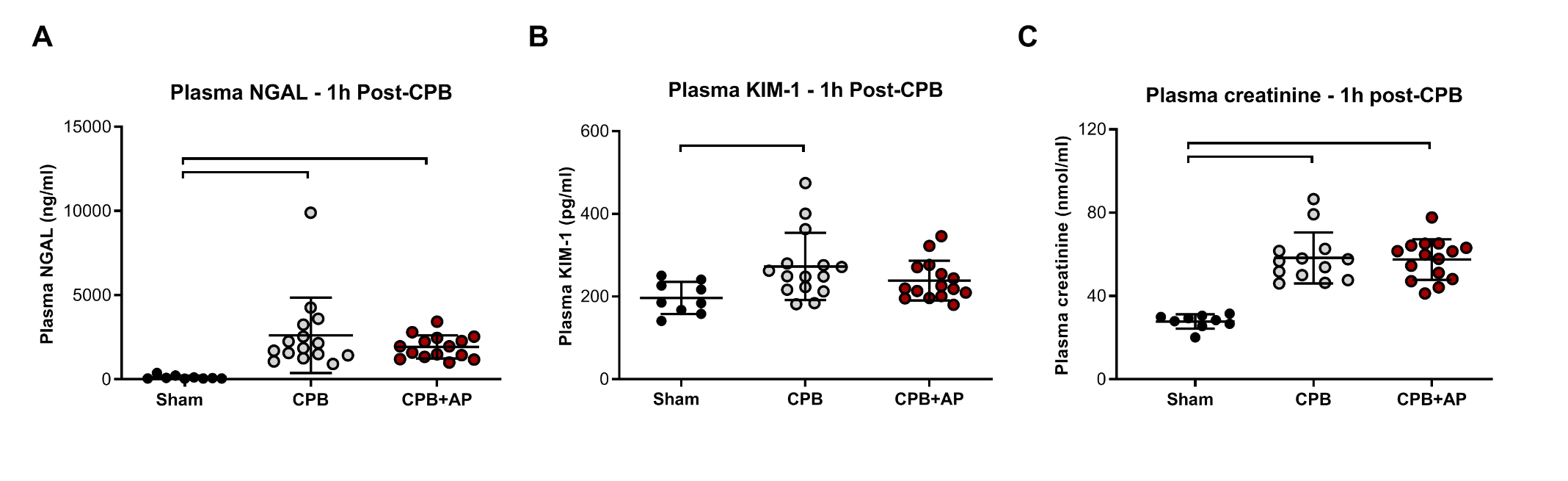
*

**Figure S3.** Plasma concentrations of renal injury molecules NGAL (**A**), KIM-1 (**B**), and creatinine (**C**) measured 1 hour (1h) after weaning from CPB. Data are presented as mean ± standard deviation. A black bar represents p < 0.05 CPB vs sham. CPB, cardiopulmonary bypass; NGAL, neutrophil gelatinase-associated lipocalin; KIM-1, kidney injury molecule-1.
